# Sestrin2 Silencing Exacerbates Cerebral Ischemia/Reperfusion Injury by Decreasing Mitochondrial Biogenesis through the AMPK/PGC-1α Pathway in Rats

**DOI:** 10.1038/srep30272

**Published:** 2016-07-25

**Authors:** Lingyu Li, Lina Xiao, Yanghao Hou, Qi He, Jin Zhu, Yixin Li, Jingxian Wu, Jing Zhao, Shanshan Yu, Yong Zhao

**Affiliations:** 1 Department of Pathology, Chongqing Medical University, Chongqing, People’s Republic of China; 2 Institute of Neuroscience, Chongqing Medical University, Chongqing, PR China; 3 Department of Pathophysiology, Chongqing Medical University, Chongqing, People’s Republic of China

## Abstract

Sestrin2 (Sesn2) exerts neuroprotective properties in some neurodegenerative diseases. However, the role of Sesn2 in stroke is unclear. The AMP-activated protein kinase/peroxisome proliferator-activated receptor γ coactivator-1α (AMPK/PGC-1α) pathway plays an important role in regulating mitochondrial biogenesis, which helps prevent cerebral ischemia/reperfusion (I/R) injury. Here, we aimed to determine whether Sesn2 alleviated I/R damage by regulating mitochondrial biogenesis through the AMPK/PGC-1α signaling pathway. To be able to test this, Sprague-Dawley rats were subjected to middle cerebral artery occlusion (MCAO) for 1 h with Sesn2 silencing. At 24 h after reperfusion, we found that neurological deficits were exacerbated, infarct volume was enlarged, and oxidative stress and neuronal damage were greater in the Sesn2 siRNA group than in the MCAO group. To explore protective mechanisms, an AMPK activator was used. Expression levels of Sesn2, p-AMPK, PGC-1α, NRF-1, TFAM, SOD2, and UCP2 were significantly increased following cerebral I/R. However, upregulation of these proteins was prevented by Sesn2 small interfering RNA (siRNA). In contrast, activation of AMPK with 5′-aminoimidazole-4-carboxamide riboside weakened the effects of Sesn2 siRNA. These results suggest that Sesn2 silencing may suppress mitochondrial biogenesis, reduce mitochondrial biological activity, and finally aggravate cerebral I/R injury through inhibiting the AMPK/PGC-1α pathway.

Stroke, which leads to adult disability, is one of the main causes of neurodegeneration and death in most developed countries. Cerebral ischemia/reperfusion injury (I/R), which is of great importance among all types of stroke, is a complex pathophysiological process. During all types of pathophysiology, enhanced levels of reactive oxygen species (ROS) and apoptosis in neuronal cells are considered very significant^1^.

Sestrin2 (Sesn2) is an important member of the Sestrin family, which is a group of highly conserved proteins that are induced by environmental stresses, including DNA damage, oxidative stress, and hypoxia^2^,^3^. It has been well established that Sesn2 is a powerful free radical scavenger. However, the exact mechanism underlying the role of Sesn2 in ROS metabolism during cerebral I/R remains largely unexplored.

Experimental evidence indicates that mitochondria are the major organelles that produce ROS within cells. The ROS-detoxifying system and mitochondrial biogenesis may play vital roles as endogenous protective mechanisms in cerebral ischemia^4^,^5^. Some researchers have suggested that increased mitochondrial biogenesis could exert neuroprotection^6^,^7^. Previous studies have demonstrated that the peroxisome proliferator-activated receptor γ coactivator-1α (PGC-1α) is a powerful stimulator of mitochondrial biogenesis and gene transcription in the liver, heart, and skeletal muscle as well as in neurological diseases^8^. PGC-1α activation or overexpression could be used to compensate for neuronal mitochondrial loss in neurodegenerative diseases in which mitochondrial dysfunction and oxidative stress injury play crucial roles in pathogenesis^9^,^10^. Recent studies have elucidated that oxidative stress and the redox state of ischemic neurons also involve PGC-1α^11^. In addition, researchers have found that mRNA and protein levels of PGC-1α were reduced in AMP-activated protein kinase (AMPK)-deficient HUVECs (human umbilical-vein endothelial cells), suggesting that AMPK is an upstream regulator for PGC-1α^12^. Furthermore, it is well known that Sesn2 is of great importance in activating AMPK^13^. However, no studies have explored a possible relationship between Sesn2, AMPK, and PGC-1α.

Therefore, we hypothesized that Sesn2 enhanced mitochondrial biogenesis and alleviated brain injury by eliminating ROS through the Sesn2-AMPK-PGC-1α pathway. To explore the protective role of Sesn2 in ischemic injury, we administered a Sesn2 small interfering RNA (siRNA) in an experimental middle cerebral artery occlusion (MCAO) model. Moreover, to demonstrate the specific protective mechanism, 5′-aminoimidazole-4-carboxamide riboside (AICAR), a well-known AMPK activator was used. We found that during cerebral I/R injury, Sesn2 was upregulated, followed by upregulation of p-AMPK, PGC-1α, and downstream factors. These effects were prevented following Sesn2 knockdown. However, under conditions of Sesn2 knockdown, AICAR treatment restored I/R injury-induced increases in expression of p-AMPK, PGC-1α, and downstream genes, finally improved neurological function, decreased cerebral infarct volume, and alleviated oxidative stress.

## Materials and Methods

### Animals and groups

Adult male Sprague-Dawley rats (N = 167) weighing 250–300 g were bred and housed at the Experimental Animal Center of Chongqing Medical University. Rats were randomly assigned to the following groups: Sham-injured (n = 20); MCAO (n = 37, 7 died); MCAO + scramble siRNA (n = 36, 6 died); MCAO + Sesn2 siRNA (n = 51, 11 died); MCAO + Sesn2 siRNA + AICAR (n = 23, 3 died). All experiments were conducted in accordance with the National Institutes of Health “Guide for the Care and Use of Laboratory Animals” and were approved by the Institutional Animal Care and Use Committee of Chongqing Medical University, Chongqing, China.

### Administration of Sesn2 siRNA and AMPK activator

Sesn2 siRNA (The sense primer 5-GCGAGAUCAACAAAUUACUTT-3 and antisense primer 5-AGUAAUUUGUUGAUCUCGCTT-3) was designed and chemically synthesized by GenePharma Corporation, Shanghai, China. Scramble siRNA, which has the same nucleotide composition of the target gene siRNA with no sequence homology to any known rat genes was used as the control. Rats were anesthetized with 3.5% chloral hydrate (350 mg/kg, ip) and placed in a stereotaxic apparatus (Taimeng Software, Chengdu, China). AICAR (Acadesine) (S1802), an activator of AMPK was purchased from Selleck, USA. 5 μl AICAR (10 μg/μl) or siRNA (2 μg/μl) was slowly injected into the left lateral ventricle over a 20-min duration using a Hamilton microsyringe with the coordinates of 1.0 mm posterior to the bregma, 2.0 mm lateral to the midline, and 3.5 mm ventral to the surface of the skull under the guidance of a stereotaxic instrument. The injection rate was 0.5 μl/min. After injection, the needle was held in place for 5 min and then removed slowly over 2 min. 24 h after the injection of siRNA, MCAO model was performed and AICAR was injected 30 min before MCAO model performed.

### Cerebral I/R model

Transient focal cerebral ischemia was introduced into rats by left MCA occlusion technique according to our previous methods^14^. In brief, a 4–0 monofilament nylon suture (Beijing Sunbio Biotech Co Ltd, Beijing, China) with a rounded tip was inserted into the left internal carotid artery through the common carotid artery stump and gently advanced to occlude the MCA. After 60 min of MCAO, the suture was removed to restore blood flow. The reperfusion was confirmed by laser Doppler (Periflux System 5000, Perimed AB, Stockholm, Sweden). The same procedure was performed on sham-operated rats, but the MCA was not occluded. Rats that did not show neurological deficits after reperfusion (neurological score <1) were excluded from the study, as well as animals that died after ischemia induction. Rats that showed neurological deficits immediately after reperfusion (neurological score >0) but were found to be experiencing skull base or subarachnoid hemorrhage were also excluded from the study. Core body temperatures were monitored with a rectal probe and maintained at 37 °C during the whole procedure.

### Evaluation of neurological deficit

Neurological deficit scores were evaluated by an examiner blinded to the experimental groups after 24 h of reperfusion. The deficits were scored on a modified scoring system developed by Longa *et al.*^15^, as follows: 0, no neurological deficits; 1, failure to extend right forepaw fully; 2, circling to right; 3, falling to right; 4, did not walk spontaneously and has depressed levels of consciousness. The higher the neurological deficit score, the more severe the impairment is of motor motion injury. Brains from these rats were analyzed for infarct volume, histological assessment, oxidative stress analysis, western blot, and real-time qPCR.

### Assessment of infarct volume

After 24 h of MCAO, the rats were anesthetized, killed by decapitation, and brains were rapidly removed and sliced into 2-mm-thick coronal sections in an adult rat brain matrix (RWD, Shenzhen, China). The slices were stained with 2% 2,3,5-triphenyltetrazolium chloride (TTC, Sigma, USA) at 37 °C for 20 min in the dark. After that washed with normal saline and then subsequently fixed in 4% paraformaldehyde at 4 °C for 24 h. Normal tissue stained red, and infarct tissue did not stain (appeared white). Each slice was photographed with a digital camera (Nikon, D5100) and analyzed using an Image J (ver1.37c, NIH). To compensate for brain edema, the corrected volume was calculated using the following equation: Percentage hemisphere lesion volume (% HLV) = [total infarct volume − (lef**t** hemisphere volume − right hemisphere volume)]/right hemisphere volume ×100%.

### Tissue preparation and histological assessment

After neurological examination, rats were anesthetized with 3.5% chloral hydrate (350 mg/kg) 24 h after reperfusion, and brains were perfused transcardially with 0.9% sodium chloride followed by 4% paraformaldehyde. After decapitation, brains were removed and immersed in paraformaldehyde for 24 h. After that, brains were dehydrated with automatic tissue hydroextractor and embedded in paraffin. Then, 5-μm coronal sections 1.20 mm anterior to and 3.6 mm posterior to the bregma were stained with 0.1% cresyl violet (Nissl staining) or hematoxylin–eosin (HE staining) according to standard protocols and prepared for subsequent microscopic mounting. Intact neurons were counted in tenfields for each section under a light microscope (400×). The cell count in the border zone around the ischemic core were obtained from same group, averaged and expressed as cell number/mm^2^ 16.

### Determination of superoxide dismutase (SOD) and malondialdehyde (MDA) levels

SOD and MDA levels were measured using commercially available assay kits (Nanjing Jiancheng Bioengineering Institute, Nanjing, China). According to the manufacturers’ instructions, the samples were rinsed, weighed, and then homogenized in 9 volumes of 9 g/L ice-cold saline. After centrifuged at 4000 rpm/min for 10 min at 4 °C, supernatant was collected. SOD activity was measured by the hydroxylamine method. Absorbance was determined at 550 nm by spectrometry. The MDA level was measured by the thiobarbituric acid method. Absorbance was measured at 532 nm by spectrometry. The protein concentration of the supernatant was determined by the Enhanced BCA Protein Assay Kit (Beyotime, Jiangsu, China).

### Western blot analysis

The brains of rats were removed rapidly after saline perfusion and rinsed in 0.9% normal saline (4 °C) to wash away the blood and blood clot. And then parts of cortex were homogenized in RIPA buffer. Lysate was centrifuged at 12000 g at 4 °C for 20 min, and the supernatant was collected. The protein concentration was estimated by the Enhanced BCA Protein Assay Kit (Beyotime, Jiangsu, China). Equal amount of protein was separated by sodium dodecyl sulfate polyacrylamide gel electrophoresis (SDS-PAGE) and then transferred onto nitro-cellulose membranes (Millopore, CA, USA). The membranes were blocked in 5% non-fat milk containing 20 mM Tris-HCl, pH 7.6, 137 mM NaCl, 0.1% Tween-20 (TBS-T) and then incubated at 4 °C overnight with primary antibodies against Sesn2(1:500, Proteintech, USA), AMPK(1:1000, Proteintech, USA), phospho-AMPK (1:500, Bioworld, USA), PGC-1α (1:1000, Abcam, Cambridge, MA, USA), NRF-1(1:500, Bioworld, USA), TFAM (1:1000, Bioworld, USA), SOD2 (1:2000, Bioworld, USA), UCP2 (1: 500, Bioworld, USA), cytochrome c(1:1000, Abcam, Cambridge, MA, USA), AIF(1:1000, Santa Cruz, Dallas, TX, USA) and β-actin(1:10000, Bioworld, USA). After that, it was incubated with HRP-conjugated secondary antibody (1:5000) for one hour at room temperature. The blotted protein bands were visualized by enhanced chemiluminescence (ECL). The density of bands was quantified using NIH Image J, and the data were normalized to β-actin.

### Quantitative real-time PCR (qPCR)

Total RNA of cortex in each group was purified by RNAiso Plus reagent (TaKaRa Biotechnology, Dalian, China) according to the manufacturer’s instructions. Reverse-transcription reaction was performed using PrimeScript RT reagent Kit With gDNA Eraser (Perfect Real Time) (TaKaRa Biotechnology, Dalian, China) to synthesize cDNA. qPCR was performed on a Bio-Rad CFX-96 Connect Real-Time System (Bio-Rad, Foster City, CA, USA) using iTaqTM Universal SYBR Green supermix (BIO RAD). The primer sequences and amplicon sizes are listed in Table 1. The data were analyzed using Bio-Rad CFX Manager software (version 2.0). The expression levels of the genes were normalized to β-actin.

The optimized qPCR assays were carried out with 5 μl of iTaqTM Universal SYBR Green supermix, 2 μl of cDNA, 2 μl forward primer and reverse primer (10 μM), and 1 μl of RNase-free water in a total volume of 10 μl, making the final primer concentration 0.1 μM. Amplification began with an initial incubation at 95 °C for 30 s followed by 40 cycles of denaturation at 95 °C for 5 s and annealing and extension at 60 °C for 30 s. A melting curve procedure was added to the Bio-Rad CFX96 Connect Real-Time System to analyze the specificity of the products. Nontemplate controls containing all reagents except cDNA was included in each PCR run. These controls generated C (t) >40 in all experiments.

### Statistical analysis

Data was expressed as mean ± standard error of the mean. Analysis was performed using GraphPad Prism software. Statistical differences between two groups were analyzed using Student’s unpaired, two-tailed t-test. Multiple comparisons (without rating scale data) were statistically analyzed with one-way analysis of variance (ANOVA) followed by Student-Newman-Keuls test. The neurological deficit scores were analyzed using the Mann-Whitney U test. A probability level of less than 0.05 was considered statistically significant.

## Results

### Knockdown of Sesn2 exacerbated neurological deficits and increased cerebral infarct volume following focal brain ischemia

**Figure 1:**
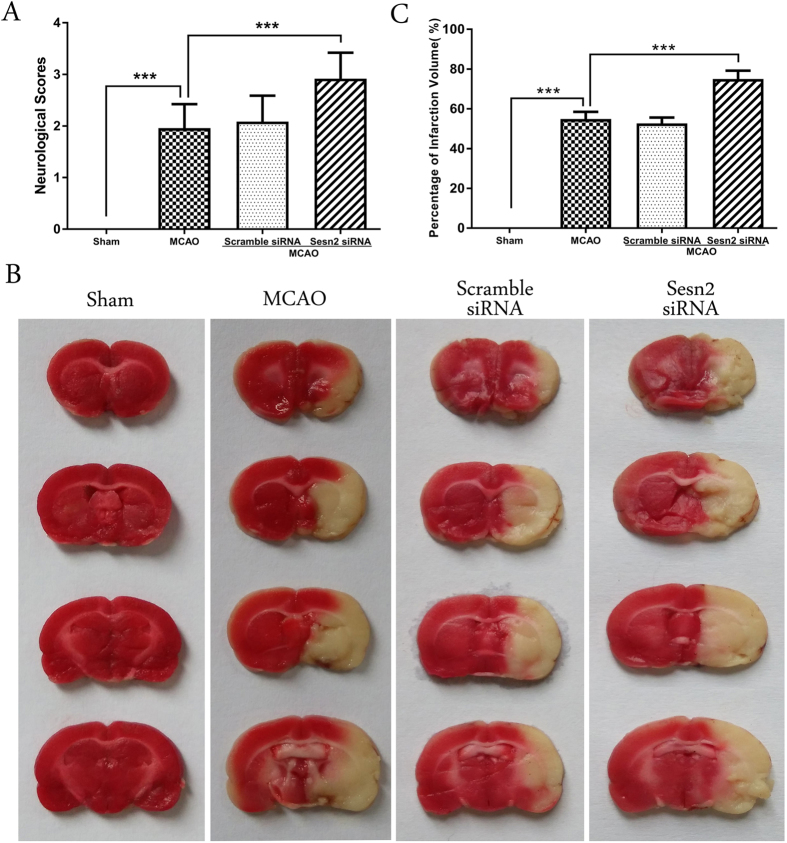
Effects of Sesn2 siRNA on neurological deficits and brain infarct volume. (**A**) Neurological scores were much higher after silencing Sesn2 than in the MCAO group. (**B**) Infarct volume in the Sesn2 siRNA group was larger than in the MCAO group. Error bars represent mean ± SEM. (***p < 0.001). Panel A n = 20 in the sham group, n = 30 in the other groups; Panel B n = 4 in the sham group, n = 6 in the other groups.

Neurological scores were assessed 24 h after I/R. Pronounced neurological deficits were observed in the MCAO rats. Compared with the MCAO group, neurological scores were increased after injection with Sesn2 siRNA (*p* < 0.001, Fig. 1A). To confirm the neuroprotective role of Sesn2, TTC staining was used to visualize and quantify infarct volumes. As shown in Fig. 1B, no infarction was observed in the sham group. The infarct volume in the Sesn2 siRNA group was significantly larger than that in the MCAO group (Sesn2 siRNA: 75.48 ± 1.83% versus MCAO: 55.58 ± 1.42%, *p* < 0.001). There was no significant difference in neurological deficits and infarct volume between the MCAO and scramble siRNA groups (*p* > 0.05).

### Sesn2 siRNA aggravated neuronal damage and oxidative stress 24 h after I/R

**Figure 2:**
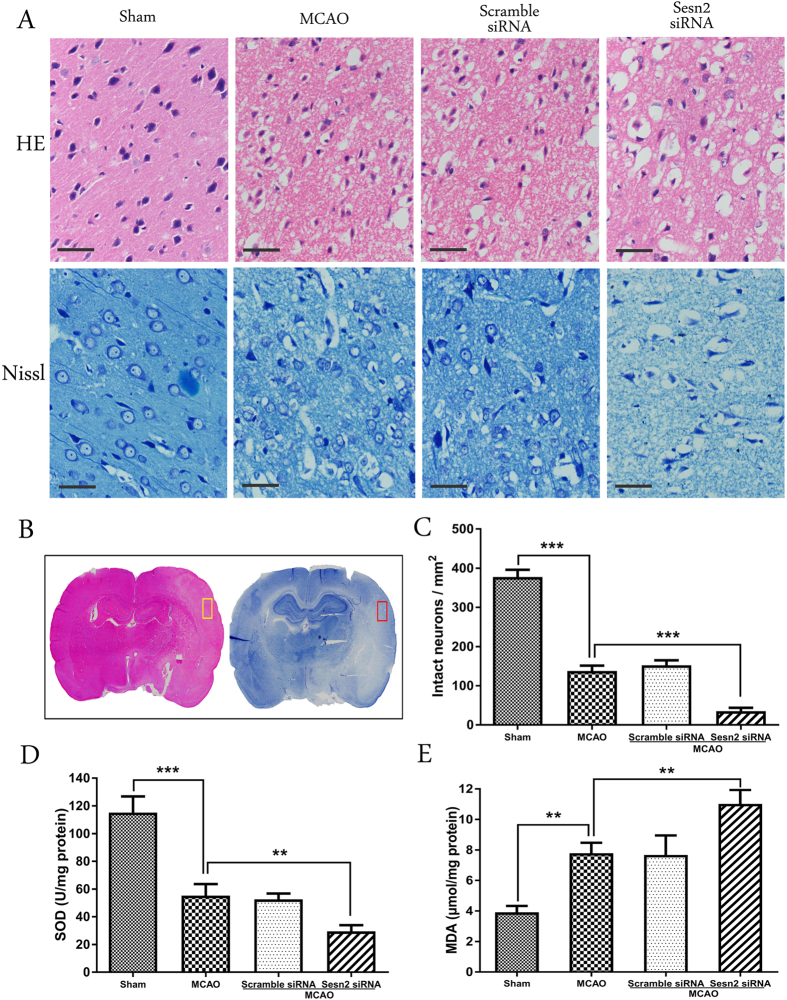
Histological assessment and oxidative stress level 24 h after focal cerebral I/R injury following Sesn2 silencing in rats. (**A**) Representative HE staining and Nissl staining in the cortex (×400). (**B**) Areas about HE and Nissl staining (in the box). (**C**) Histograms showing quantification of intact neurons in the cortex. (**D**) SOD activity was lower in the Sesn2 siRNA group than in the MCAO group. (**E**) Histograms showing that MDA levels in the Sesn2 siRNA group were significantly higher than in the MCAO group. Scale bars = 50 μm. Error bars represent mean ± SEM. (**p < 0.01; ***p < 0.001). n = 4 in the sham group, n = 6 in the other groups.

HE and Nissl staining were used to assess morphological changes 24 h after reperfusion (Fig. 2A). The area examined was shown in Fig. 2B. HE staining showed that, compared with neurocyte in the sham group, neurocyte in the MCAO and scramble siRNA groups displayed a disorderly arrangement with loosened cytoplasm and karyopyknosis. However, the Sesn2 siRNA group showed diffuse vacuolization and edema in the interstitial spaces and a decrease in the number of intact neurocyte (Fig. 2C). Similar results were obtained with Nissl staining, which showed that there were no morphological changes of neurons in the sham group. In contrast, many atrophic neurons with shrunken cytoplasm and damaged nuclei were observed in the MCAO and scramble siRNA groups. Sesn2 siRNA increased the number of degenerated neurons, and most neurons showed loss of Nissl bodies (*p* < 0.001).

Oxidative stress was examined by measuring SOD activity and MDA content in ischemic cortical tissue. A significant decrease in SOD activity was observed in rats subjected to I/R (*p* < 0.001, Fig. 2D). Animals injected with Sesn2 siRNA showed a further reduction of SOD activity compared with the MCAO group (29.51 ± 2.54 versus 55.19 ± 4.85 U/mg, *p* = 0.0076). The results of MDA content analysis are shown in Fig. 2E. Compared with the sham group, animals in the MCAO group showed significant increases in MDA levels in the ischemic cortex (7.78 ± 0.39 versus 3.91 ± 0.24 μmol/mg, *p* = 0.0013). Furthermore, the MDA level was greater in the Sesn2 siRNA group than in the MCAO group (11.01 ± 0.53 versus 7.78 ± 0.39 μmol/mg, *p* = 0.0042).

### Downregulation of Sesn2 suppressed p-AMPK and PGC-1α expression following transient MCAO, but the effect was reversed using an AMPK activator

**Figure 3:**
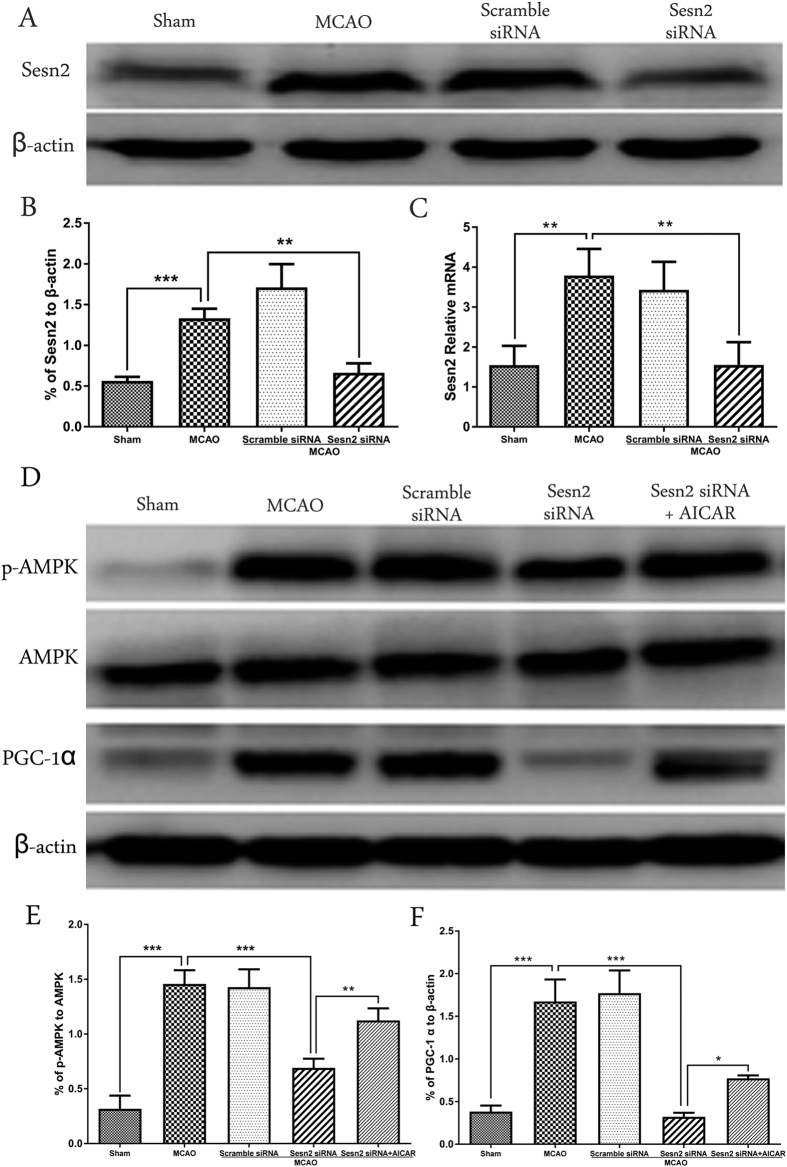
Representative western blots and quantitative analysis of Sesn2 (**A**), p-AMPK, AMPK, PGC-1α (**D**), qPCR results of Sesn2 (**C**). Relative protein band density values were calculated as the ratio of protein of interest to that of β-actin. Quantification of (**A**,**D**) is shown in (**B**,**E**,**F**) respectively. Error bars represent mean ± SEM. (*p < 0.05; **p < 0.01; ***p < 0.001). n = 4 in the sham group, n = 6 in the other groups.

To explore the impact of Sesn2 on expression levels of p-AMPK and PGC-1α, western blot and qPCR were performed (Fig. 3). After cerebral I/R, expression of Sesn2 was upregulated approximately 2.6-fold at the protein level (*p* = 0.0010) and 2-fold at the mRNA level (*p* = 0.0040), followed by upregulation of p-AMPK and PGC-1α. Sesn2 siRNA decreased expression of Sesn2 (*p* = 0.0023), as well as expression of p-AMPK (*p* < 0.001) and PGC-1α (*p* < 0.001) (Fig. 3A,D). However, the effects of Sesn2 siRNA on p-AMPK and PGC-1α were reversed by AICAR (p-AMPK: *p* = 0.0046; PGC-1α: p = 0.0270, Fig. 3D).

### Sesn2 siRNA decreased expression of mitochondrial biogenesis proteins, NRF-1 and TFAM, as well as ROS-detoxifying proteins, SOD2 and UCP2, and this effect was prevented by AICAR

**Figure 4:**
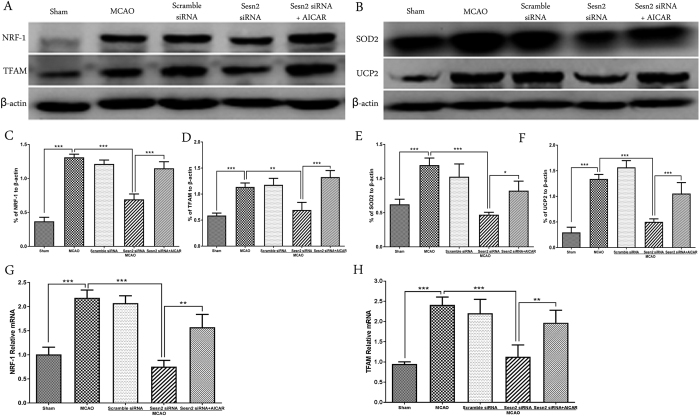
Representative western blots and quantitative analysis of NRF-1 and TFAM (**A**), SOD2 and UCP2 (**B**). Histograms showing the qPCR results of NRF-1 (**G**) and TFAM (**H**). Relative protein band density values were calculated as the ratio of protein of interest to that of β-actin. Quantification of (**A**,**B**) is shown in (**C**–**F**) respectively. Error bars represent mean ± SEM. (*p < 0.05; **p < 0.01; ***p < 0.001). n = 4 in the sham group, n = 6 in the other groups.

Protein and gene expression of NRF-1, TFAM, SOD2, and UCP2 were analyzed by western blot and qPCR, respectively (Fig. 4). In the MCAO group, protein levels and gene expression of NRF-1 and TFAM significantly increased (*p* < 0.001), accompanied by increases in SOD2 and UCP2 expression (*p* < 0.001). Sesn2 siRNA prevented this up-regulation and reduced expression of all of these factors (All *p* < 0.001 except TFAM: p = 0.0018). However, under Sesn2 silencing conditions, after AICAR was administered, expression of these factors increased again following MCAO (NRF-1 and TFAM: *p* < 0.001; SOD2: p = 0.0151; UCP2: 0.0012).

### Knockdown of Sesn2 enhanced levels of mitochondria-related apoptosis proteins, but levels were reduced after administering AICAR

**Figure 5:**
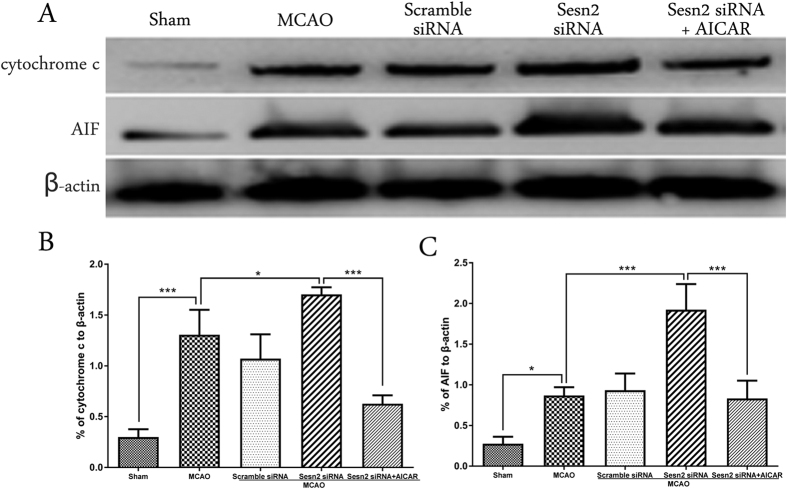
Representative western blots and quantitative analysis of cytochrome c and AIF. Relative protein band density values were calculated as the ratio of protein of interest to that of β-actin. Quantification of (**A**) is shown in (**B**,**C**) respectively. Error bars represent mean ± SEM. (*p < 0.05; ***p < 0.001). n = 4 in the sham group, n = 6 in the other groups.

Cytochrome c and AIF are 2 key mitochondria-related apoptogenic factors. Western blot analysis showed that expression of cytochrome c and AIF was induced following cerebral I/R (cytochrome c: *p* < 0.001; AIF: *p* = 0.0152, Fig. 5). After Sesn2 had been silenced, expression of both factors was even higher (cytochrome c: *p* = 0.0421, AIF: *p* < 0.001). In contrast, treatment with AICAR decreased protein levels of cytochrome c and AIF after I/R (*p* < 0.001).

### Activation of AMPK with AICAR reduced Sesn2 siRNA induced neurological deficits, cerebral infarct volume and oxidative stress

**Figure 6:**
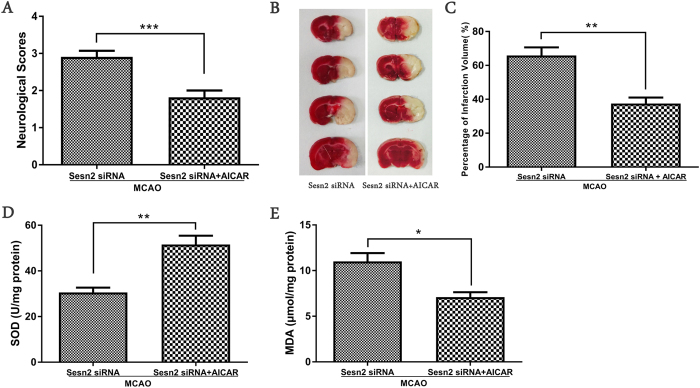
Activation of AMPK with AICAR reduced Sesn2 siRNA induced neurological deficits, cerebral infarct volume and oxidative stress. (**A**) Neurological scores in the Sesn2 siRNA group and Sesn2 siRNA + AICAR group at 24 hours following cerebral I/R. (**B**) Cerebral infarct volume in the Sesn2 siRNA group and Sesn2 siRNA + AICAR group at 24 hours following cerebral I/R. (**C**) SOD activity in the Sesn2 siRNA group and Sesn2 siRNA + AICAR group. (**D**) Histograms showing the MDA levels in the Sesn2 siRNA group and Sesn2 siRNA + AICAR group. Error bars represent mean ± SEM. (*p < 0.05; **p < 0.01; ***p < 0.001). Panel A: n = 10 per group; Panel (C–E): n = 5 per group.

Twenty-four hours after AICAR injection with Sesn2 silencing, neurological scores were significantly improved (*p* < 0.001), cerebral infarct volume was obviously diminished (*p* = 0.0032), SOD activity was enhanced (*p* = 0.0030) and MDA content was decreased (*p* = 0.0205) compared to rats only injected with Sesn2 siRNA (Fig. 6).

### AICAR applied alone further increased expressions of effectors in the AMPK/PGC-1α pathway

**Figure 7:**
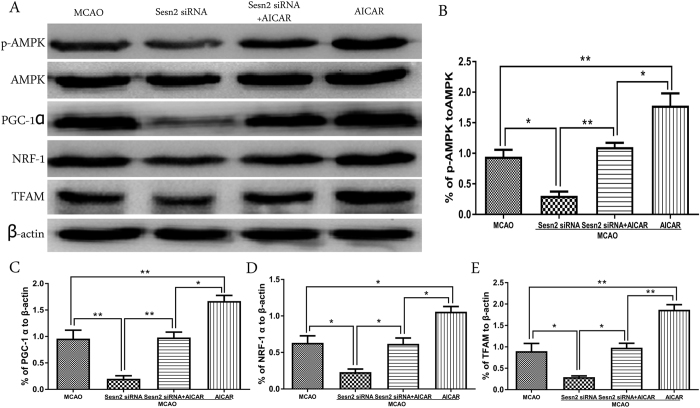
Representative western blots p-AMPK, AMPK, PGC-1α, NRF-1 and TFAM. Relative protein band density values were calculated as the ratio of protein of interest to that of β-actin. Quantification of (**A**) is shown in (**B**–**E**) respectively. Error bars represent mean ± SEM. (*p < 0.05; **p < 0.01). n = 4 in the sham group, n = 6 in the other groups.

To explain the impact of Sesn2 on AMPK/PGC-1α pathway more accurately, AICAR was used alone (Fig. 7). The results demonstrated that the activation of p-AMPK was further enhanced compared with that in Sesn2 siRNA+AICAR group (*p* = 0.0198). The downstream of AMPK, PGC-1α, NRF-1, and TFAM expressions were also much more increased in comparison with those injected both Sesn2 siRNA and AICAR (PGC-1α: *p* = 0.0100; NRF-1: *p* = 0.0127; and TFAM: *p* = 0.0036).

## Discussion

In the present study, we demonstrated that expression of Sesn2 was elevated in the cortex after cerebral I/R. In contrast, Sesn2 knockdown effectively eliminated I/R-induced Sesn2 elevation in brain tissues. Sesn2 silencing exacerbated neurological deficits, increased cerebral infarction, and increased oxidative stress and neuron damage. To investigate the mechanism of Sesn2 in this experiment, AICAR, an AMPK activator, was administered after Sesn2 down-regulation. Sesn2 silencing was accompanied by decreases in p-AMPK/AMPK, mitochondrial biogenesis proteins, PGC-1α, NRF-1, and TFAM, as well as ROS-detoxifying proteins, UCP2 and SOD2, and increases in mitochondria-dependent apoptotic factors, cytochrome c and AIF. However, these effects were prevented by AICAR. These results suggest that Sesn2-induced AMPK-PGC-1α activation may be one underlying mechanism of the neuroprotective roles of Sesn2 against brain injury following cerebral I/R.

Oxidative and nitrosative stress are powerful mediators of ischemic injury. After cerebral ischemia, the balance between ROS production and clearance are compromised, which may result in oxidative stress-induced signaling and cell injury^17^. Sesn2 is an inducible protein that protects cells against exaggerated ROS levels. One study reported that Sesn2 was induced in human glioblastoma U87 cells following ionizing radiation (IR). Sesn2 silencing not only increased oxidative stress but also sensitized U87 cells to IR^18^. Another study suggested that silencing Sesn2 in renal proximal tubule cells increased hyperoxidized peroxiredoxins and ROS production^3^. Recent studies showed that Sesn2 played a pivotal role in age-dependent neurodegenerative diseases. Sesn2 expression was elevated in primary rat cortical neurons upon Aβ exposure and in the cortices of a mouse model of Alzheimer’s disease^19^. Nevertheless, the potential involvement of Sesn2 in ischemia has not been well studied. In our experiment, Sesn2 expression was elevated approximately 2.6-fold after cerebral I/R. However, Sesn2 knockdown exacerbated neurologic deficits and increased cerebral infarction. Moreover, Sesn2 elimination decreased SOD activity and increased levels of MDA product after MCAO. These results demonstrated the neuroprotective role of Sesn2.

The mechanisms Sesn2 exerts its protective effects against oxidative damage in cerebral ischemia remain unclear. It may promote the activity of other oxidoreductases, such as peroxiredoxin (Prdx), sulfiredoxin (Srx), that also regenerate Prdx^20^,^21^. However, another study showed that Sesn2 is not a reductase for cysteine sulfinic acid of peroxiredoxins^20^. Till now, the antioxidative mechanism of Sesn2 is not sure. It was accepted that ROS were generated principally by mitochondria during ischemia. Mitochondria are also the sources of energy and metabolism^6^. Several recent studies have shown that ROS could mediate mitochondrial biogenesis^22^. Another study has shown that excessive ROS may cause oxidative damage to mitochondria and elicit cell apoptosis. Cytochrome c and AIF are 2 key mitochondria-related apoptosis genes^23^. Therefore, maintaining the biogenesis of mitochondria and respiratory functions is pretty important^24^. Mitochondrial biogenesis is a highly regulated process and occurs on a regular basis in healthy cells, where it is controlled by the nuclear genome. It has been demonstrated that several protein factors encoded by nuclear genes are involved in the biogenesis of mitochondrial and respiratory functions, including NRF-1, TFAM, and PGC-1α^25^,^26^. It has been shown that PGC-1α may be the initiating factor of mitochondrial biogenesis^27^,^28^. Similar to Sesn2, PGC-1α is activated under oxidative stress as well. It has been reported that PGC-1α is expressed in the mouse cerebral subcortex under hypobaric hypoxia^29^,^30^. Silencing PGC-1α expression in mice exacerbates the neurodegenerative effects of 1-methyl-4-phenyl-1,2,3,6-tetrahydropyridine (MPTP) and oxidative stressors in the substantia nigra and hippocampus. In contrast, increasing PGC-1α levels dramatically protect neural cells in culture from oxidative stressor-mediated death^10^,^31^. It was reported that PGC-1α is required for induction of many ROS-detoxifying proteins, including UCP2 and SOD2^32^. PGC-1α activity is enhanced by interacting with NRF-1 through the NRF-1 DNA binding domain. In neuronal cells, PGC-1α activation or overexpression promotes expression and activation of NRF-1, which regulates expression of downstream TFAM and other nuclear-e ncoded genes associated with oxidative phosphorylation to promote mitochondrial biogenesis^33^. It has also been reported that reperfusion increased mitochondrial biogenesis by increasing PGC-1α, NRF-1 and TFAM following focal cerebral ischemia^22^. In our experiment, after cerebral I/R, we observed an increase in expression of PGC-1α followed by increased expression of NRF-1, TFAM, SOD2 and UCP2.

Several signaling kinases have been implicated in mediating PGC-1α transcriptional activation in response to various stimuli. The most important is AMPK. Pharmacological activation of AMPK with AICAR markedly increases PGC-1α and NRF-1 mRNA levels^34^. In a global cerebral ischemia rat model, AMPK activity was up-regulated and involved in induction of mitochondrial biogenesis proteins PGC-1α, NRF-1, and TFAM. Inhibition of AMPK by compound C reversed these effects, further supporting the role of AMPK upstream of mitochondrial biogenesis proteins^35^. Through direct physical association as well as indirect transcriptional regulation, Sesn2 is able to bind AMPK and induce formation of the AMPK holoenzyme and its phosphorylation and activation by upstream kinases such as LKB1^13^,^36^. A recent report also demonstrated that, through AMPK activation, Sesn2 can inhibit nicotinamide adenine dinucleotide phosphate (NADPH) oxidase 4 (NOX4), which generates pathogenic levels of cytosolic ROS^37^.

The mechanism through which Sesn2 regulates mitochondrial function is still unclear and few studies have been reported. With the preceding discussion, Sesn2 likely protects cerebral tissue from ROS injury by activating the AMPK signal pathway, which then up-regulates expression of mitochondrial biogenesis proteins PGC-1α, NRF-1, and TFAM. Our results support this hypothesis. After 24 h of reperfusion, Sesn2 was significantly increased, and AMPK activity was enhanced accompanied by its downstream effectors PGC-1α, NRF-1 and TFAM. Expression of mitochondrial ROS-detoxifying proteins UCP2 and SOD2 was also dramatically increased. In contrast, with Sesn2 silenced, AMPK activity was remarkably down-regulated and down-regulation of PGC-1α, NRF-1, and TFAM. UCP2 and SOD2 expressions were also reduced. Nevertheless, all of these effects of siRNA were reversed after administering the AMPK activator, AICAR. However, compared with administrated both Sesn2 siRNA and AICAR, AICAR applied alone much more increased the AMPK activity and the expression of PGC-1α, NRF-1 and TFAM. This could be due to the inhibition of AMPK after silencing of Sesn2. Furthermore, under Sesn2 silencing conditions, the neurological deficits were improved, cerebral infarct volume was diminished, and oxidative stress was reduced in rats injected with AICAR. These results reconfirmed our speculation.

## Conclusions

The present study provided novel evidence that Sesn2 exerted neuroprotective effects during cerebral I/R injury, possibly by increasing mitochondrial biogenesis. Sesn2 silence suppressed AMPK activation, leading to down-regulation of PGC-1α and downstream signaling factors, which results in decreased mitochondrial biogenesis and enhanced cerebral oxidative stress. These findings may provide a new therapeutic target in the development of treatments for cerebral ischemic damage.

## Additional Information

**How to cite this article**: Li, L. *et al.* Sestrin2 Silencing Exacerbates Cerebral Ischemia/Reperfusion Injury by Decreasing Mitochondrial Biogenesis through the AMPK/PGC-1α Pathway in Rats. *Sci. Rep.* **6**, 30272; doi: 10.1038/srep30272 (2016).

## Figures and Tables

**Table 1:** Information on the qPCR primers.

Gene symbol	GenBank accession no.	Primer sequence (5′to 3′)	Product size(b.p.)
Sesn2	NM_001109358.2	GGCTGTGGGATACTTCCTGA	104
		TTCAATGGGTCTCTGCTTGG	
NRF1	NM_001100708.1	CGAAAGAGACAGCAGACACG	122
		AAGACAGGGTTGGGTTTGG	
TFAM	NM_031326.1	CAGAGTTGTCATTGGGATTGG	137
		TTCAGTGGGCAGAAGTCCAT	
